# Digital twin-driven prognostics and health management for industrial assets

**DOI:** 10.1038/s41598-024-63990-0

**Published:** 2024-06-11

**Authors:** Bin Xiao, Jingshu Zhong, Xiangyu Bao, Liang Chen, Jinsong Bao, Yu Zheng

**Affiliations:** 1 School of Mechanical Engineering, Shanghai Jiao Tong University, Shanghai, 200240 China; 2 College of Mechanical Engineering, Donghua University, Shanghai, 200240 China

**Keywords:** Digital twin (DT), Fault, Industrial assets, Prognostics and health management (PHM), Engineering, Mechanical engineering

## Abstract

As a facilitator of smart upgrading, digital twin (DT) is emerging as a driving force in prognostics and health management (PHM). Faults can lead to degradation or malfunction of industrial assets. Accordingly, DT-driven PHM studies are conducted to improve reliability and reduce maintenance costs of industrial assets. However, there is a lack of systematic research to analyze and summarize current DT-driven PHM applications and methodologies for industrial assets. Therefore, this paper first analyzes the application of DT in PHM from the application field, aspect, and hierarchy at application layer. The paper next deepens into the core and mechanism of DT in PHM at theory layer. Then enabling technologies and tools for DT modeling and DT system are investigated and summarized at implementation layer. Finally, observations and future research suggestions are presented.

## Introduction

Prognostics and health management (PHM) research and applications have expanded from aerospace to manufacturing, energy, transportation, and many other fields. Proper maintenance reduces downtime and production costs, and improves operational efficiency and production. PHM optimizes the maintenance work of industrial assets from complex industrial equipment and its components to large-scale production systems such as production lines and workshops.

Fault detection, diagnosis, prediction, etc., work as responsive parts of PHM when there are faults or potential faults in industrial assets^1^. By responding promptly to faults, researchers optimized system availability and ensured production continuity and stability by several detail aspects, i.e., fault monitoring, detection, isolation, identification, diagnosis, prediction, and handling^2^, which are explained in Table 1. Artificial intelligence-based approaches have been widely developed in these aspects. Such data-driven approaches rely on data models to deal with faults. However, complex equipment and mass production systems often involve multiple components and machines with intricate operational processes and interconnections. This complexity hinders data models from accurately capturing the relationship between faults and system responses, resulting in imprecise fault analysis. Meanwhile, the disturbing factors from the external environment and the variability in operating conditions pose challenges to the generalizability and robustness of the data model in practical scenarios^3^.

**Table 1:** Terminology in the paper^6^.

Term	Definition
Fault	Anomaly that a system's inability to a specified function
Fault mode	Fault type reflects the macroscopic behavior of a system
Fault cause	Key factors leading to a fault
Fault mechanism	Physical process changes that give rise to a fault
Fault feature	Feature or parameter indicating the anomaly caused by a fault
Fault monitoring	Process of tracking and observing the object’s status and performance
Fault detection	Process of determining whether a fault has occurred
Fault isolation	Process of determining the fault type and/or location
Fault identification or estimation	Process of determining the fault cause and the fault magnitude or intensity
Fault diagnosis	Process of detecting, isolating, and estimating a fault
Fault prediction	Process of identifying if a fault will occur in future
Fault handling	Process of taking measures to restore normalcy

Digital twin (DT) leverages cutting-edge technologies like artificial intelligence, cloud computing, and big data analytics to create multi-dimensional DT model. DT model integrates diverse data sources, such as geometric features, physical attributes, sensor monitoring, and expert knowledge, to enable precise virtual mapping of physical assets^4^. Based on an integrated technologies and data view, DT model comprehensively reflects various characteristics and states of industrial assets across the operational cycles. By the fusion analysis of multiple data sources, DT model can reveal hidden patterns and correlations behind the data. Accordingly, clearer insights of fault mechanisms and propagation paths can be derived to enable deep understanding and accurate analysis of faults^5^. Therefore, the application of DT in PHM can enhance the comprehensiveness, depth, accuracy, and interpretability of fault analysis, providing optimized support for the operation and maintenance of industrial assets.

**Figure 1:**
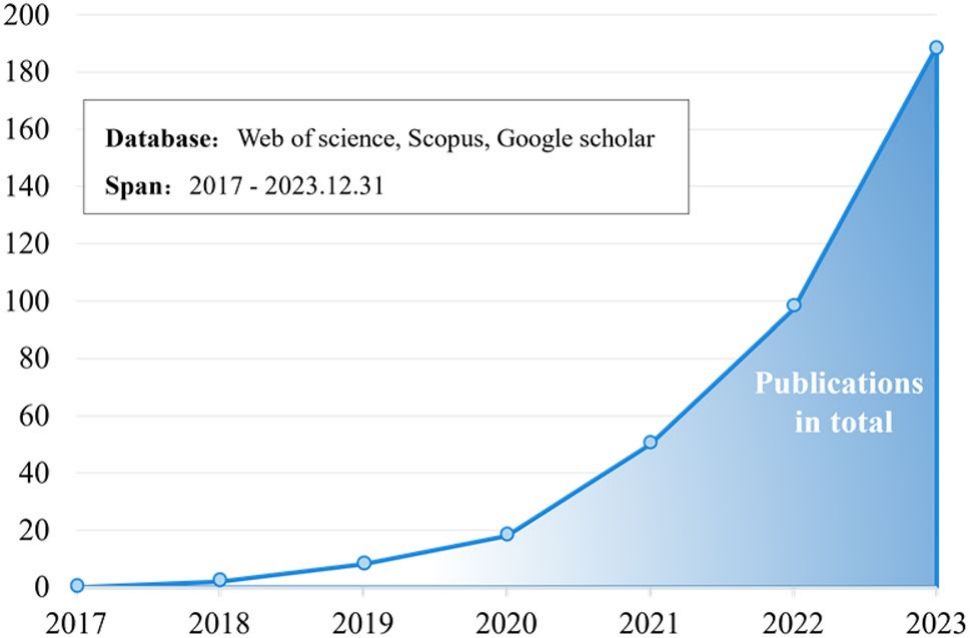
Growing trend of DT-PHM studies for faults of industrial assets.

**Figure 2:**
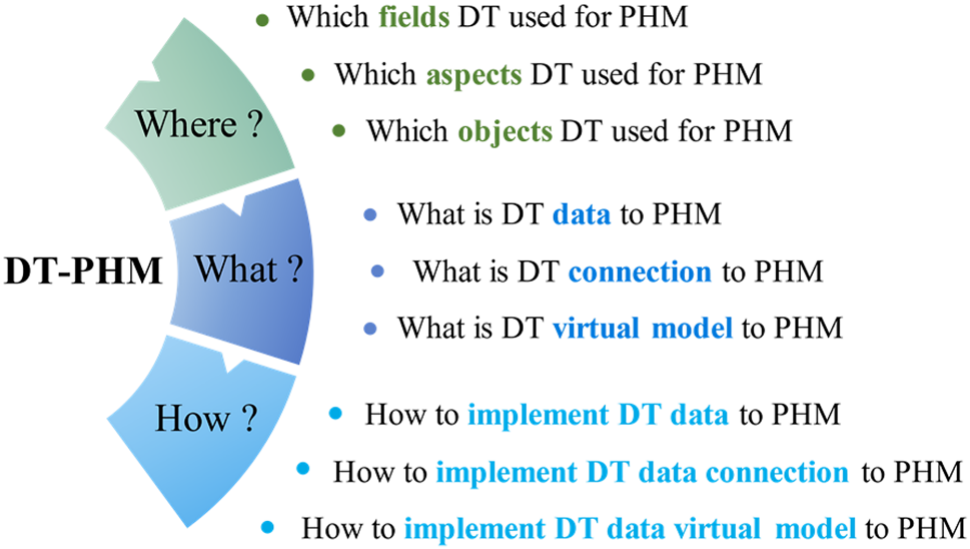
Research questions of this paper.

As a result, DT is widely used to facilitate PHM research. In current DT-driven PHM (DT-PHM) research, studies focused on the faults of industrial assets is rapidly rising in recent years with increasing publications and growing influence, as presented in Fig. 1. However, comprehensive analysis and summary of DT used in PHM from insights on industrial asset faults are unavailable. Thus, this paper provides a systematic study for DT-PHM research by addressing the following three research questions (RQs) from application layer, theory layer, and implementation layer. Figure 2 specifies the RQs.

1. Where applies DT in PHM?
2. What underpins DT in PHM?
3. How implements DT in PHM?

### Related review works

DT-driven PHM research is a hot topic with lots of published academic publications, including some reviews. Cui et al.^7^ overviewed theoretical concepts related to DT-PHM studies for electromechanical products. Khalid et al.^8^ further reviewed data-based and model-based approaches and explored their correlations in DT-PHM applications for aircraft structure. Yüce et al.^9^ provided a technical overview of the role of machine learning techniques in the DT-driven PHM methods for wind energy infrastructure.

These papers provided insightful overview works of DT-driven PHM studies from theoretical concepts, and methodologies to technical details for specific industrial objects. The objective of DT-driven PHM research is to ensure the healthy operations of industrial assets. However, as a key factor that directly affects the operation, the fault is not fully considered in these reviews. The lack of fault analysis of industrial assets may confine the research to theoretical discussions, hampering the real-world applicability and effectiveness of DT-PHM technologies.

For DT-PHM reviews targeting faults of industrial assets, only five are currently available for limited objects or scenarios. These reviews introduced the fault detection of building assets^10–12^ fault prediction of wind turbine^13^, and abnormality information management of optical communication process^14^.

In fact, current DT-PHM research has expanded to various aspects like diagnosis, prediction, and handling, to address faults in industrial objects at different hierarchical levels in various application fields. A systematic and comprehensive review work is not reachable for DT-driven PHM research with a spotlight on the faults of industrial assets.

The research gaps for current DT-PHM studies are summarized as follows:

- So far, there has been no review paper studying DT in PHM from insights on the faults of industrial assets to systematically investigates DT-PHM from application, theory, and implementation.
- In application layer, comprehensive summaries and analysis of DT applications in PHM from multiple perspectives are not available.
- In theory layer, core elements of DT model and their collaboration which determine the DT effectiveness in PHM, are currently not well investigated in existing studies.
- In implementation layer, no framework exists to guide the practical implementation of DT theories for PHM from enabling technologies and tools, as well as DT systems.

### Contributions of this paper

To bridge research gaps and address research questions, this paper systematically analyzes and summarizes DT-PHM studies from insights on the faults of industrial assets, making the following core contributions:

- Provide a comprehensive summary of DT applications in PHM by the application field, aspect, and hierarchy to clarify RQ1 “Where applies DT in PHM?” at application layer of DT-PHM.
- Investigate the data, connection, and virtual model of DT model in PHM to reveal the core and mechanism behind DT and answer RQ2 “What underpins DT in PHM?” at theory layer of DT-PHM.
- Establish a systematic framework of the enabling technologies and tools of DT data and connection modeling, virtual model construction, as well as DT systems for PHM and explain RQ3 “How implements DT in PHM?” at implementation layer of DT-PHM.

The rest of this paper is organized as follows. Section "Literature review method and research framework" describes review method and framework. Section "DT application in PHM" analyzes DT applications in PHM. Section "DT model to PHM" studies the core elements and their collaboration of DT in PHM. Section "DT modeling tech, tool, and DT system for PHM" investigates DT implementation in PHM. Section "Challenges and opportunities" presents challenges and opportunities. Lastly, Section "Conclusions and future work" concludes the paper.

## Literature review method and research framework

### Literature review method

**Figure 3:**
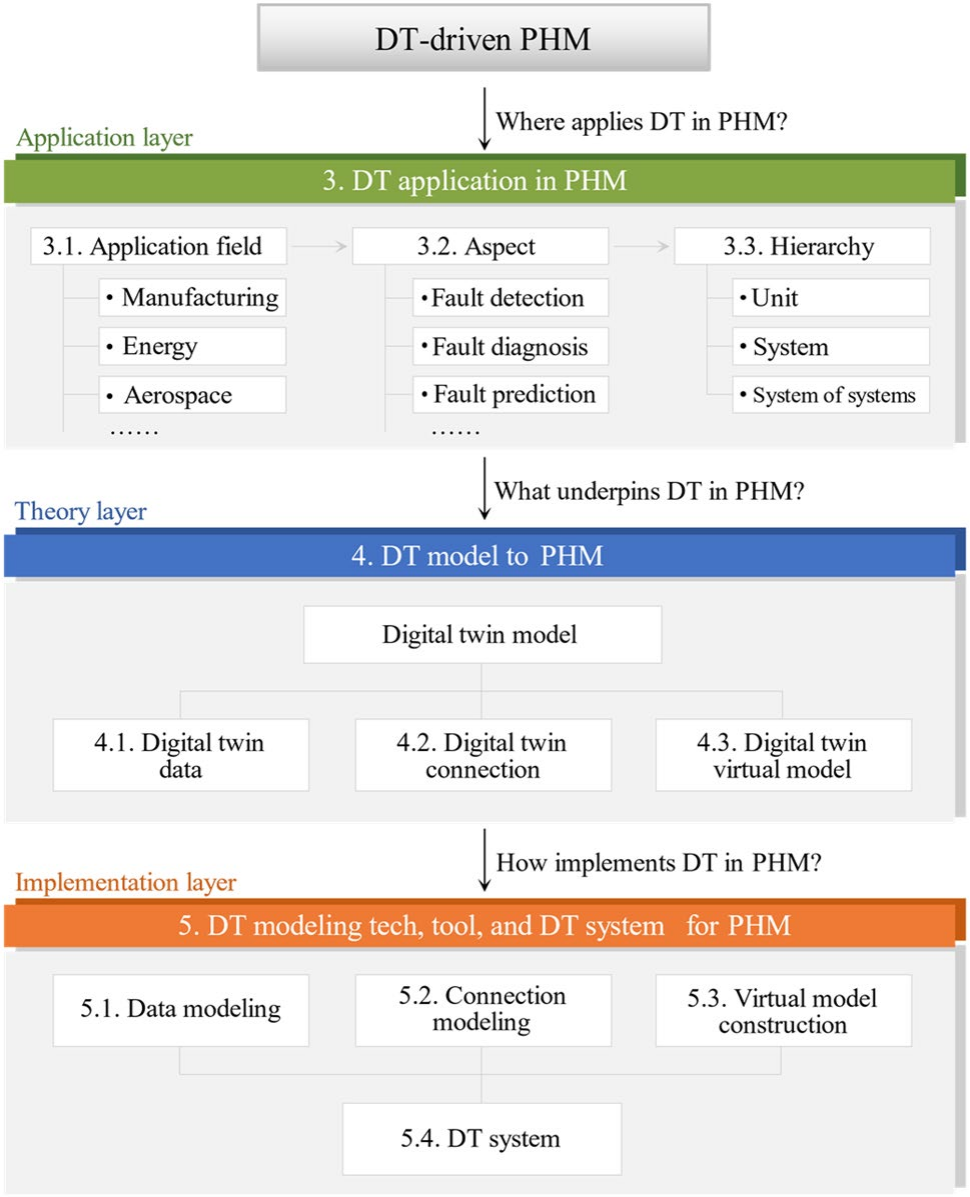
Research framework of this paper.

The review work is grounded in scientific publications including journal articles, conference papers, book chapters, reviews, and editorials. Our review method follows a three-step process: (1) Retrieve the leading databases by advanced document search with specific query strings to obtain the original retrieved documents. To ensure the comprehensiveness, keywords such as “anomaly”, “failure”, and “malfunction” are included in title retrieval besides “fault”, and wildcard “*” is also used for approximate phrases such as “fault”, “faults”, and “faulty”. (2) Remove irrelevant publications by reviewing detailed content. The relevance of each retrieved publication to the research themes (e.g., DT model attributes for fault diagnosis) is evaluated. For example, despite the keywords “digital twin” and “fault” included in the publication title, its study focused on how to construct a DT model by the method of fault trees instead of research or approach to address faults. Such irrelevant publications are removed. (3) Review the entire content of all relevant publications selected in step 2 and analyze them by the research framework presented in Fig. 3. Table 2 presents the retrieval indexes in the above process.

**Table 2:** Retrieval index of literature review.

Retrieval index	Detail content
Database	Web of science, Scopus, Google scholar
Query strings	Digital twin* AND (Fault* OR Failure* OR Anomal* OR Abnormal* OR Glitch* OR Malfunction* OR Breakdown*)
Document retrieved	189
Papers selected	167
Time scope	2003—2023.12.31

## Research framework

The research framework of this paper, presented in Fig. 1, clarifies the three RQs raised in Introduction by reviewing the selected literature. For RQ1, section "DT application in PHM" comprehensively studies the application field, aspect, and hierarchy of DT in DT-PHM studies at application layer to explain in which application field and for which object in different hierarchies, which aspect of PHM was carried out through DT. The DT model, synchronizing with physical entity in virtual space, serves as the carrier of DT functions and the basis for services^15^. Therefore, for RQ2, section "DT model to PHM" discloses the core and mechanism of DT in DT-PHM studies at theory layer from DT data, connection, and virtual model, which are key elements of DT model. Accordingly, the adoption of enabling technologies and tools for DT modeling goes beyond theories and facilitates the effective implementation of DTs in practical scenarios. Furthermore, DT model and modeling are encapsulated into services by DT systems to achieve intelligent equipment diagnosis, product quality tracing, production troubleshooting, etc., in practical applications. Hence, for RQ3, section "DT modeling tech, tool, and DT system for PHM" explores enabling technologies and tools of DT modeling from data and connection modeling, and virtual model construction, as well as related DT systems of DT-PHM studies at implementation layer to guide DT implementation in industrial scenarios.

## DT application in PHM

**Figure 4:**
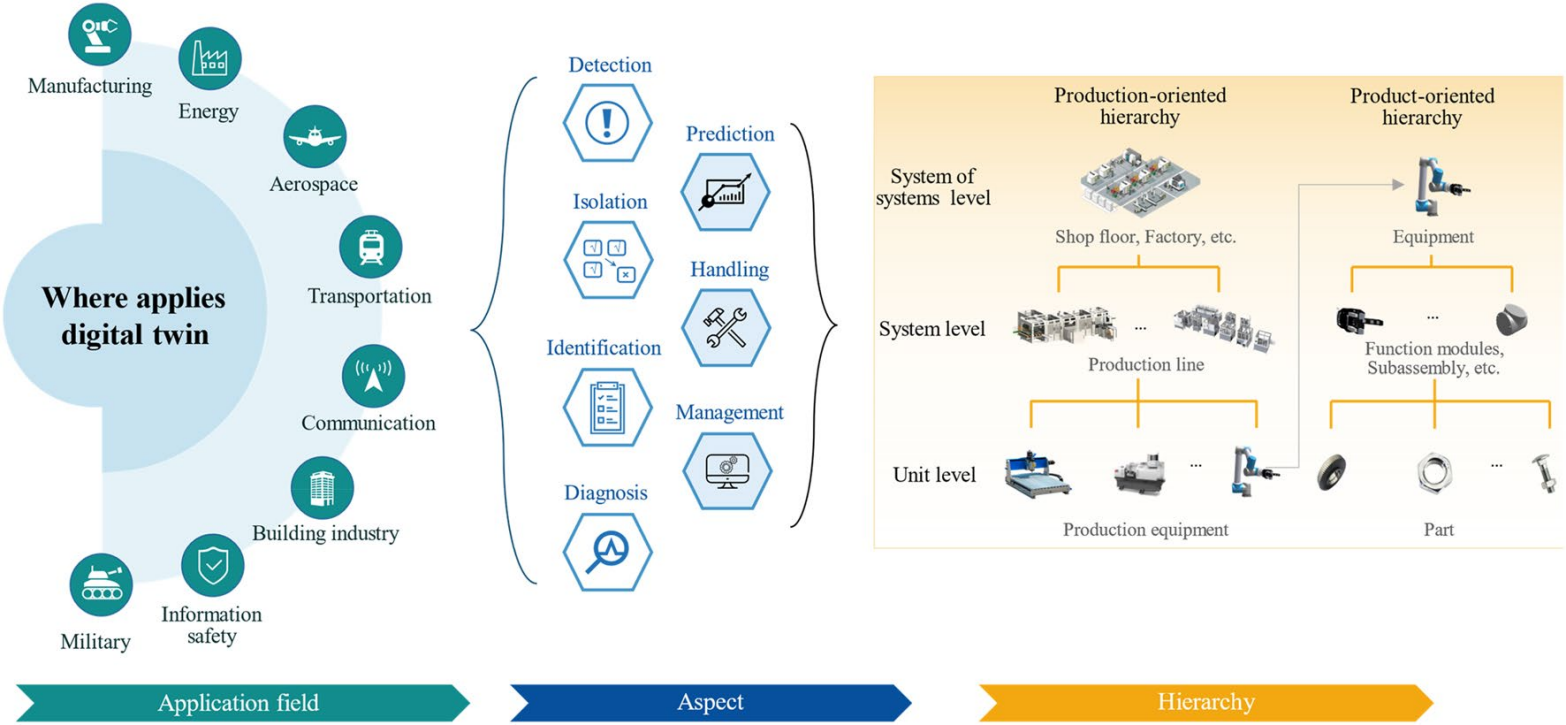
DT application in PHM.

This section analyzes the DT application in PHM at application layer of DT-PHM to answer RQ1 “Where applies DT in PHM?”. Application fields are summarized first. Then the different aspects, including fault detection, diagnosis, prediction, etc., are investigated in related application fields. Finally, from different hierarchical levels of industrial assets, we analyze research objects in the application field whose faults were diagnosed or predicted. RQ1’s research organization is presented in Fig. 4. Later sections detail the study with statistics.

### Application fields

**Figure 5:**
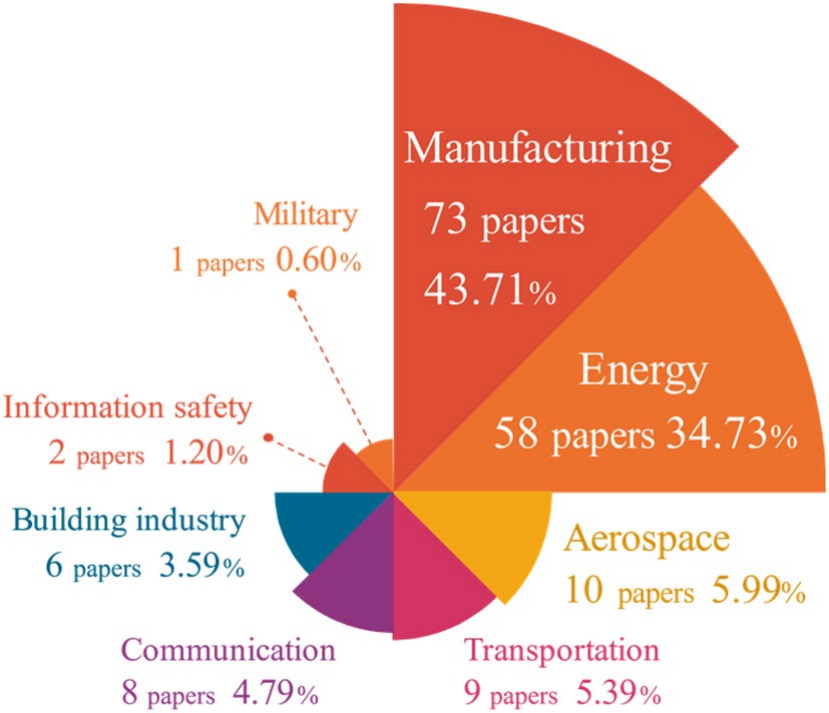
Application field distribution of DT-PHM.

Figure 5 presents the 167 selected papers which are classified into 8 application fields according to their target applications in case studies. The percentage of each field corresponds to the respective fan area totaling 100%. In DT-PHM studies, manufacturing remains the focus of DT, with nearly half of the reviewed papers. The research in this area is both broad in scope and detailed in its approach. From the bearing as the basic unit of mechanical transmission, to the joints that support and connect the production actions of the equipment, as well as the production equipment for specific production tasks, their fault studies have been performed from multiple elements of DT, including diverse types of data, different dimensions of virtual model, and connections between them, etc. Studies are also extended to the manufacturing process of production line and shop floor. Based on multi-perspective analysis of various application objects in manufacturing, the services including operational status monitoring, abnormal production warning, and maintenance recommendations, etc., can be accessible by enabling technologies and tools, and developed DT systems. However, the individuality and distribution diversity of production units challenges the integration and deployment of DT modeling technologies for fault monitoring in virtual space. The complex manufacturing environment with uncontrollable factors and variables rises difficulties in maintaining the accuracy of DT models in fault diagnosis either. Therefore, transfer learning^16–24^ is a popular approach for DT-PHM to enhance the generalizability of DT models in real manufacturing scenarios.

The energy sector stands as the second hot area for DT used in PHM, with a primary focus on energy-related equipment, components, and energy production processes. Energy facilities interacts multiple physical fields like temperature, pressure, and flow. DT modeling requires the coupling of model variables of these fields to quantify fault impact and propagation paths. Around 22% of publications were distributed in other 6 fields, including aerospace, communication, transportation, etc. Nowadays, DT has been applied in at least 18 domains and many more sub-domains^18^ like electric vehicle (EV)^25^ and unmanned aerial vehicle (UAV) industries^26^. For PHM in industrial assets, DT application is limited. Meanwhile, manual experience is still relied upon in areas where faults are a concern^27^. For example, fault monitoring and prediction can be performed by DTs of the EV and UAV. Existing or potential faults are analyzed in DT virtual models. Based on the analytical feedback from the virtual-reality mapping mechanism, fault prevention and handling can be facilitated in the physical EV and UAV. DT-PHM in such promising fields is a big potential for both academic research and application.

### Aspects

**Figure 6:**
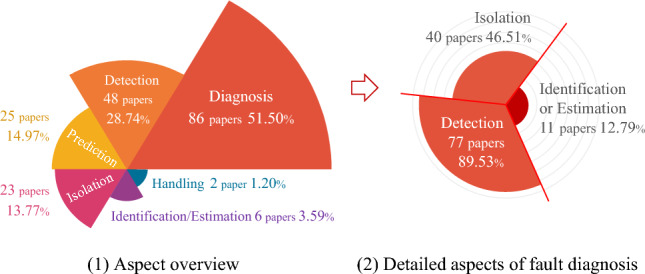
Research aspect distribution of DT-PHM.

Based on the data acquired from the fault monitoring, the reviewed publications utilized DT frameworks, methodologies or approaches to enhance fault research in six aspects, i.e., fault detection, isolation, identification, prediction, as well as handling, as shown in Fig. 6. Because fault monitoring is the base for PHM development, the statistical analysis focuses on the above six research aspects. One publication may explore PHM from multiple research aspects. These publications are counted in each relevant aspect of the fan diagrams in Fig. 6, resulting in a total percentage greater than 100%. Fault diagnosis is a hot topic and significant for practical engineering^28^. Diagnosis was also the focus of DT application in PHM. We analyze the articles on diagnosis, refining DT fault diagnosis into three detailed aspects: detection, isolation, and identification by fault analysis depth. Based on fault detection results, fault isolation and identification can be performed sequentially to locate fault and clarify causes, then impact quantification can be derived. The complete DT diagnostic process reduces physical trial and error in virtual space, resulting in lower operation and maintenance (O&M) costs of the physical assets. In DT machining practice^29^, data-driven models were utilized to detect faulty sensor data of the tool spindle, followed by mechanism models to identify the root causes and quantify their impact on production. As shown in Fig. 6, current DT diagnostic research primarily focused on the stage of detecting whether a fault has occurred or not, but neglected further fault analysis. If fault diagnosis only stops at detection stage, DT model fails to provide an effective fault response plan. This may lead to blind attempts in physical troubleshooting.

General physical assets have varying fault types and characteristics across domains, and even within the same domain, structural discrepancies exist between similar physical assets, systems, and processes. These discrepancies hinder the selection and configuration of data, algorithms, and models for PHM. Therefore, fault information management across PHM research aspects is necessary to identify historical fault cases and solutions for physical assets in same or different domains. By classification, summarization and statistics, the fault causes and patterns can be clarified to preclude failures and optimize O&M. Meanwhile, the effective management of diverse data and accumulated knowledge from multiple PHM research aspects lays the foundation for a systematic DT-PHM methodology. However, fault information management in current DT-PHM merits more attention.

### Hierarchy

**Figure 7:**
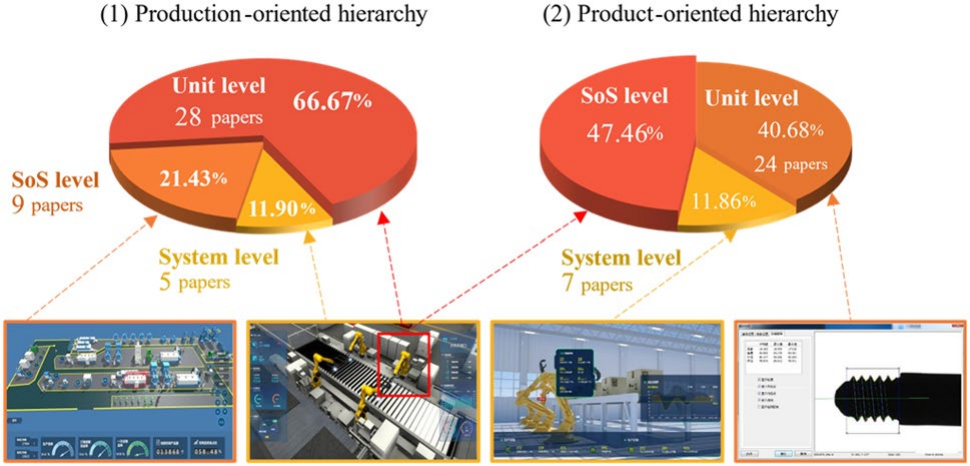
Hierarchical distribution of manufacturing.

For a clear and comprehensive profile of DT application in PHM, the industrial assets are studied from different levels of the hierarchical structure. A well-designed hierarchical structure facilitates management, coordination, and reuse of the algorithms, data, and knowledge in DT-PHM, which reduces research costs and improves DT model adaptivity across industrial assets in different hierarchies. Industrial assets in manufacturing are classified into production-oriented and product-oriented hierarchies^18^. Figure 7 visualizes the 73 reviewed papers reflecting the hierarchical theory in manufacturing. As the production unit in the production-orient hierarchy and System of Systems (SoS) in the product-orient one, equipment objects are the research focus in both hierarchies. Current literature methods were applied for fault detection of a single equipment or machine. For SoS level of production-oriented hierarchy, the trend of developing cluster manufacturing is rising^30^. By sharing experience and knowledge among similar machines in cluster manufacturing, transferable DT models can predict fault probability and impact level driven by real-time data. It provides a cost-effective way to maintain machines’ reliability and stability for cluster manufacturing. Hence, it is crucial for DT models with generalization capabilities to be easily adapted to similar industrial objects or scenarios and be adjustable to different PHM aspects. However, the model available for machine fleets challenges DT modeling with research gaps.

In DT-PHM, DT in other application fields lack hierarchical concepts, preventing the research and application from developing strategies in a macro view. As an illustration, in communication systems, functional models of sensors, transmission, and network devices are established at the hardware layer, and operational logic and protocols are expressed at the software layer. By integrating elements of each layer, a DT network is built to provide comprehensive system monitoring. To fully leverage DT benefits in PHM, an explicit hierarchical structure besides the field of manufacturing is required.

## DT model to PHM

After understanding the current status of DT application in PHM at application layer, this section will study the DT model behind these DT applications at theory layer to answer RQ2 “What underpins DT in PHM”. DT incorporates the physical system and its digital models. The physical structure, characteristics, and failure modes are expressed in virtual space by modeling. The model analyzes and processes multiple types of data to reflect parameter changes of operational states. Driven by model, DT can perform fault monitoring, detection, and diagnosis, etc., for physical system. Therefore, as function carrier and the basis for service delivery, DT model is the core of DT in PHM.

A reasonable model framework specifies the organizational structure and topology within DT model, clarifies the correlation between failures and model elements. Table 3 lists the currently recognized theoretical frameworks of DT models. Based on these theoretical frameworks, some reviewed papers constructed their DT models towards specific industrial objects or scenarios for PHM. The five-dimensional DT model framework^31^ proposed that data, connection, and virtual model comprise of the DT model. By exploiting various types of data to build multi-dimensional virtual models and establishing internal and external connections, DT model can provide high-fidelity mapping to physical entities.

**Table 3:** Available DT model frameworks.

Refs.	Feature	Gaps in fault research
^31^	Five-dimensional DT model framework	Neglected collaboration between multi-dimensional models under failure modes
^32^	General DT framework of the product	Ignored extraneous factors’ effects on model state
^33^	Reference framework of DT in manufacturing	Incomplete model expression to fault behavior and state
^34^	DT framework based on model-based systems engineering	Overlooked data variability in fault analysis
^35^	Cloud-based DT framework for healthcare	Inadequate reflection on the correlation between functional models and failures
^36^	DT framework of cyber physical production system	Rely on historical data only
^37^	DT framework for cardiology	Single application object with limited generalization
^38^	DT framework of building information modeling	Insufficient detail of dynamically varying fault features
^39^	DT framework for smart city	Unclear delineation of specific model functions for faults

**Figure 8:**
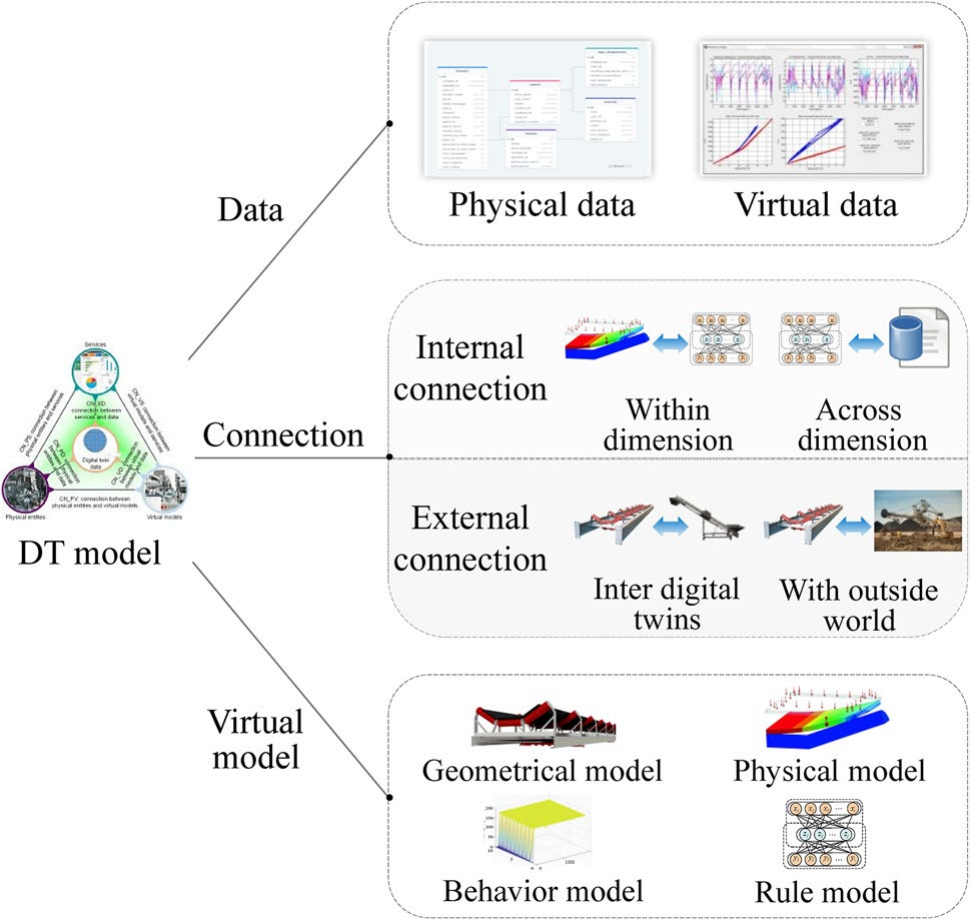
DT model to PHM.

Although model frameworks were expressed differently by respective research purposes to faults^40^, Table 3 presents that they can be further improved in terms of DT data, connection, and virtual model for PHM. Therefore, later subsections investigate the DT model from data, connection, and virtual model as shown in Fig. 8.

### DT data to PHM

**Figure 9:**
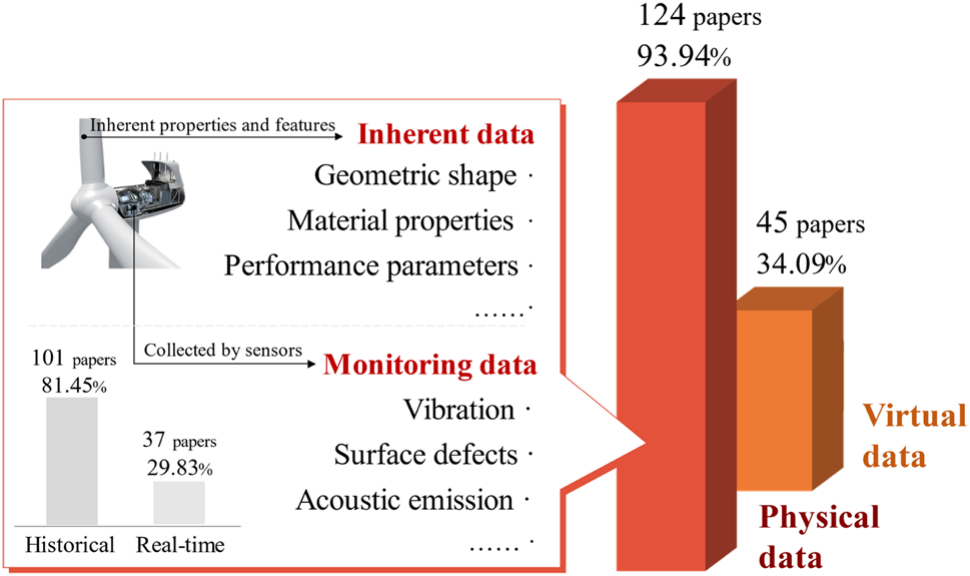
Data category of DT model in PHM.

Acquired from the physical world, physical data can be input into DT model for fault analysis of industrial assets. Driven by physical data, the DT model simulates the behavior and state of physical entities, and fault features are extracted and analyzed during the simulation to identify anomalous behaviors and predict potential fault states. Compared to physical data, the data generated by DT model simulation is virtual data, which can be used to further validate and calibrate the model by the deviation from physical entity. Accordingly, DT data in DT-PHM is categorized into physical and virtual data, which are highlighted in 132 reviewed papers as depicted in Fig. 9. Some papers applied both types of data, resulting in a bar graph sum greater than 100%. About 94% reviewed papers used physical data, including inherent and monitoring data. Inherent data is set since a physical entity is manufactured and persists with it, such as performance parameters^41^, which is the basis for DT modeling in machine fault detection. Monitoring data can be collected by various sensors, which may contain anomalous values such as outliers in vibration that reflect abnormal behaviors and states during the dynamic evolution of production equipment. By virtual-real mapping mechanism, fault features are further processed and analyzed in DT model. However, full expression of dynamically varying fault features is necessary for accurate diagnosis based on monitoring data. Poorly expressed fault features affect the diagnostic credibility of DT model, which hinders rational maintenance strategy development. Therefore, the reliability of monitoring data is crucial for the well-expressed fault features in DT-PHM studies. Accordingly, the paper^42^ provided a comprehensive and insightful overview work of studies related to the calibration and certification of industrial sensors to ensure that valid monitoring data can be provided by the sensors. To streamline sensor calibration and prevent oversight of sensor errors, an online monitoring method of sensor calibration status was also proposed to facilitate condition-based maintenance^43^. In monitoring data, only around 30% papers were real-time data driven. Real-time data enables agile feedback and adjustment of the DT model, allowing immediate response to failures in practical engineering scenarios. Hu et al.^44^ extracted the real-time signal features in each monitoring frame and fused them into the DT model for accurate fault detection of the engine. Real-time data can be used to continuously refine the failure analysis mechanism and modes of base model built by historical data, which is an advisable data-driven approach to improve fault prediction as well.

Real data acquisition can be costly, while virtual data can be a cost-saving alternative when monitoring data is insufficient^45^. Virtual data is derived from the computation and simulation of the DT model, which can be fault analysis results or extracted hidden abnormal information. 45 reviewed papers used virtual data to facilitate their research. By adjusting generating parameters, virtual data was generated under various conditions to increase the robustness and generality of the DT model^46^. In engineering scenarios, rare faulty data for training DT models is challenging because machines primarily operate normally and the most data collected is healthy^47^. In^48^, faulty conditions of the autoclave were simulated by DT models, and the generated faulty data was used to improve fault prediction.

In addition, some reviewed articles used public datasets, which reduced the time and cost of data collection and labeling, and accelerated the development of DT models. In fact, public data may not be applicable to real production environments. However, it can be used to test the generalization ability of the DT model in fault pattern recognition^49^.

### DT connection to PHM

DT connection enables data transmission and information exchange of DT model. DT connection includes internal and external connections.

#### Internal connection

Internal connection includes connections within and across dimension. Within-dimension connections include those between sub-models in the virtual model or different data types^50^. Connection between the virtual model and data is across dimension. By connecting and integrating processed abnormal data and analysis results from functional models, a comprehensive understanding of failures can be achieved, providing valuable insights into fault mechanism.

1. Connection within-dimension
  By connecting sub-models, the barriers between heterogeneous models are eliminated, allowing the dynamic coupling and full expression of the geometry, physical properties, behavioral responses, and knowledge rules within DT model. Thus, collaboration between dimensional models can be realized to synthesize different failure modes. In^51^ analytical results of the functional sub-models were extracted as common features for interactive feedback between them. Algorithmic model calculated the integrated analysis of functional models to predict gearbox’s faults. Connections between types of data can be troublesome due to discrepancies in data acquisition and representation. By the logic of data interaction in DT model, a proper data processing mechanism should be established to realize the connection between different types of data. However, methodologies and practices for data fusion are inaccessible in current DT-PHM research.
2. Connection across dimension.
  Connection across dimension fuses data and models to facilitate PHM. Data-driven approaches excel with large datasets, but are susceptible to noise, anomalies, or missing data which can be addressed by model-driven approaches through the simulation of virtual models. Furthermore, the data-driven virtual model can reason and update empirical knowledge, which facilitates model parameters optimization by fusing with other data. This mutual connection between model and data enables DT model to be self-updating. Paper^52^ used machine learning model with expertise to generate novel production knowledge. The knowledge was extracted as features and integrated with sensor data to evolve the DT model of the manufacturing system. Faults that arose during the evolution were diagnosed.

#### External connection

External connections can be further classified into the connection inter DTs and the connection with outside world. Studying external connections can reveal the impact of external factors on DT model and the physical assets, thus providing more comprehensive information for failure analysis in DT-PHM approaches. Regrettably, external connections were ignored in current DT-PHM research. More specifically, what is lacking in the current DT-PHM research about external connections are (i) insufficient research on environment perception and feedback mechanism; (ii) insufficient analysis of external disturbing factors; and (iii) insufficient coupling with external systems. For (i), the lack of real-time perception and feedback mechanism to the external environment prevents DT models from capturing the impact of environmental changes on the state of physical assets in time to enable timely strategy adjustment and response. For (ii), a variety of external disturbances could impact on the operational states of physical assets. However, current research tends to limit its attention to the internal characteristics and operational data of the physical asset itself and lacks comprehensive analysis and modeling of external environmental factors. For (iii), the DT model's lack of tight integration with external systems hampers its ability to effectively collaborate with other intelligent systems or decision support systems, thus limiting comprehensive management and optimization of physical assets.

### DT virtual model to PHM

**Figure 10:**
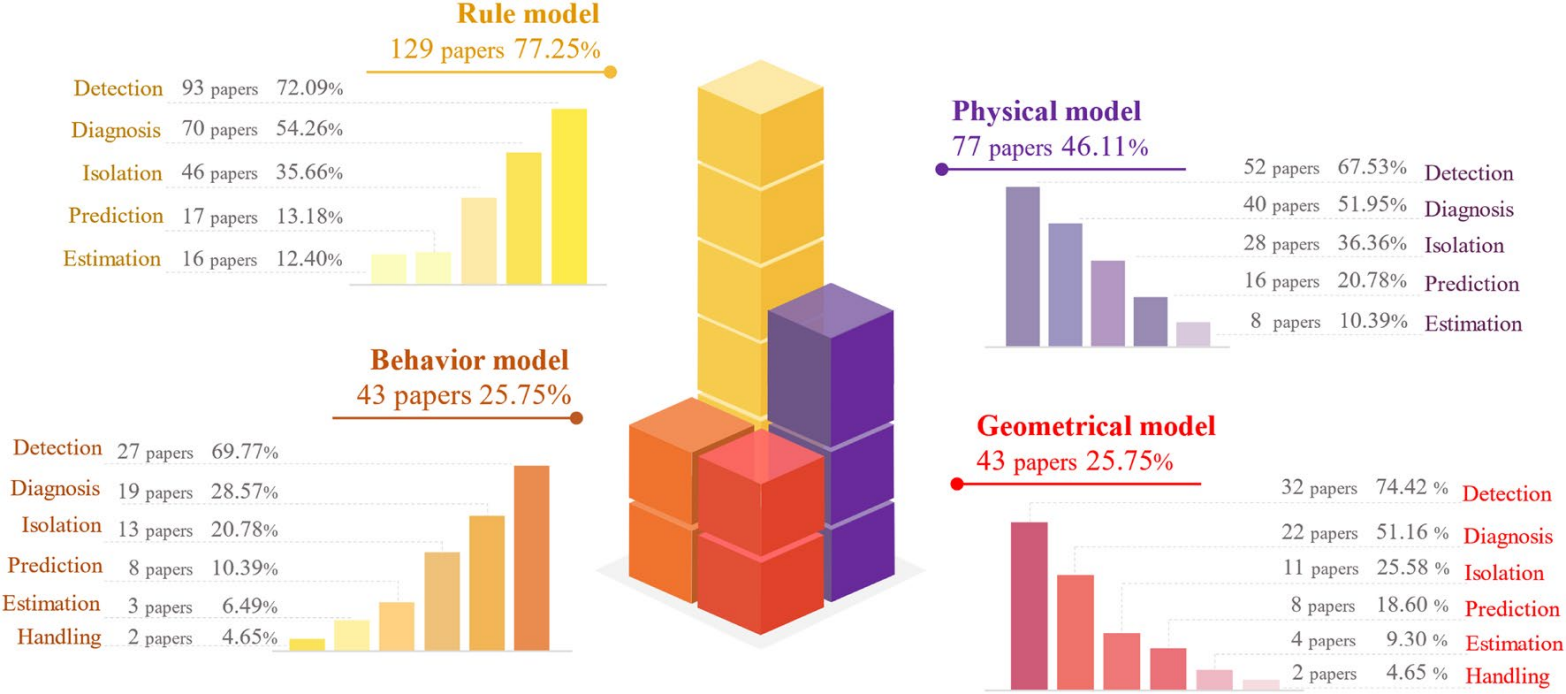
Data distribution of multiple dimensions of DT virtual model in PHM.

Driven by data and expertise, the virtual model of DT model simulates the properties, behaviors, and rules of physical entities to detect, diagnose, predict faults in virtual space. For a comprehensive and precise mapping of physical entity, the virtual model is refined into sub models in multiple dimensions, i.e., geometrics, physics, behavior, and rule, as explained in Table 4. Based on the feature, expression and functionality theorized by prior works, in-depth study and summaries of these multi-dimensional models in DT-PHM research are provided in this subsection. By analyzing the proposed methods, approaches, and effects rather than solely relying on reviewed articles’ statements to their contributions, the actual functionality and collaboration of each dimensional model in DT-PHM studies are evaluated in detail and visualized in Fig. 10. Out of 167 reviewed, papers studied faults through multiple dimensions, resulting in a total bar graph greater than 100%.

**Table 4:** Multiple dimensions of virtual model^53^.

Dimension	Feature	Expression	Functionality
Geometric model	Geometric appearance, assembly relationships	3D models, geometric parameters	Visualization
Physical model	Inherent attributes, physical constraints	Finite element model, mathematical models, topological information	Mechanism analysis
Behavioral model	Dynamic and kinematic behaviors, evolutionary behaviors, functional behaviors	Dynamic and kinematic equations, finite state machine, behavior tree	Behavior description
Rule model	Tacit knowledge generalization, data association and exploitation, rule expression	Expert systems, machine learning models, statistical models	Decision making

Each dimension of the virtual model plays a unique role in enhancing the DT effectiveness through their interconnections. As a basic part of DT, geometric model visualizes information such as motion trajectories and vibration conditions in virtual space. The visualization clarifies the working characteristics of physical entities, which enables more visualized simulation of faults and their effects. Meanwhile, geometric models can help locate where is failing faster by clustering and classification of mesh points. However, geometric models are underutilized in DT-PHM with only 28% studies.

Physical model is the core and advantage of DT-driven approaches over data-driven ones in DT-PHM studies. Physical model explains the abnormal behaviors and failure mechanisms of physical entity by mathematizing its working principle, structural characteristics, and other relevant factors. Through physical models, we can understand how and why faults occur and the physical reasons behind them. This explanatory nature enables accurate fault detection and diagnosis in depth while takes appropriate isolation action for repair. Accordingly, Fig. 10 illustrates that physical models primarily contribute to detection, diagnosis, and isolation in DT-PHM studies.

As the manifestation of dynamic attributes of DT, behavior model portrays the real-time responsive behaviors under external environment and internal operational mechanisms of the physical entity. The fault modes can be identified by the responsive behaviors. For example, the behavioral model can monitor the operational status of the equipment, where abnormal vibrations, looseness, or blockages in the production behavior of the equipment will be detectable. Figure 10 presents behavior models were less studied. Behavior modeling is challenging as it involves multiple factors and their impact on physical entity. Moreover, nonlinear characteristics and time-varying motions further complicate the modeling.

Rule model is intelligence center of DT in DT-PHM research. It exploits data correlation and tacit knowledge to infer the physical entity’s evolution. Throughout the evolutionary process, the rule model’s parameters are updated to intelligently respond to sudden abnormal changes and new failure modes. Figure 10 shows that 79% studies fed data to mine rules for intelligent failure analysis and decision-making. However, how to extract, interpret, and reuse rules obtained by scenario-specific data is distinguished from transfer learning that applies pre-trained models to tasks in new domains. New methods are required for DT-PHM research.

Instead of working alone, multi-dimensional models increase the rapidity, interpretability, and veracity of fault prediction through parallel computation, mutual corroboration, and compensation. To enhance the quality and yield of energy products, Wang et al.^48^ developed a four-dimensional virtual model for autoclave fault prediction. Geometric model described autoclave’s shape and assembly. Physical model defined key attributes, such as temperature, volume, and pressure, and integrated them into autoclave’s geometric structure. Behavioral model simulated the curing process based on autoclave's geometry and physics. Rule model mined data and fuse domain knowledge to extract rules. Faults were predicted by analyzing process curve based on the rules.

Few reviewed articles incorporated all dimensions in modeling. Current DT-PHM studies is disproportionate across model dimensions as shown Fig. 10. More papers focused on the rule and physical dimensions, with less attention paid to behavior and geometry. Inadequate model dimension is one-sided and cannot realistically map physical entities for PHM. Meanwhile, each model dimension stagnated in fault detection and diagnosis and need to be extended to more PHM research aspects. Mobilizing and integrating each model dimension to innovate fault handling is a promising avenue that has yet to be explored.

## DT modeling tech, tool, and DT system for PHM

**Figure 11:**
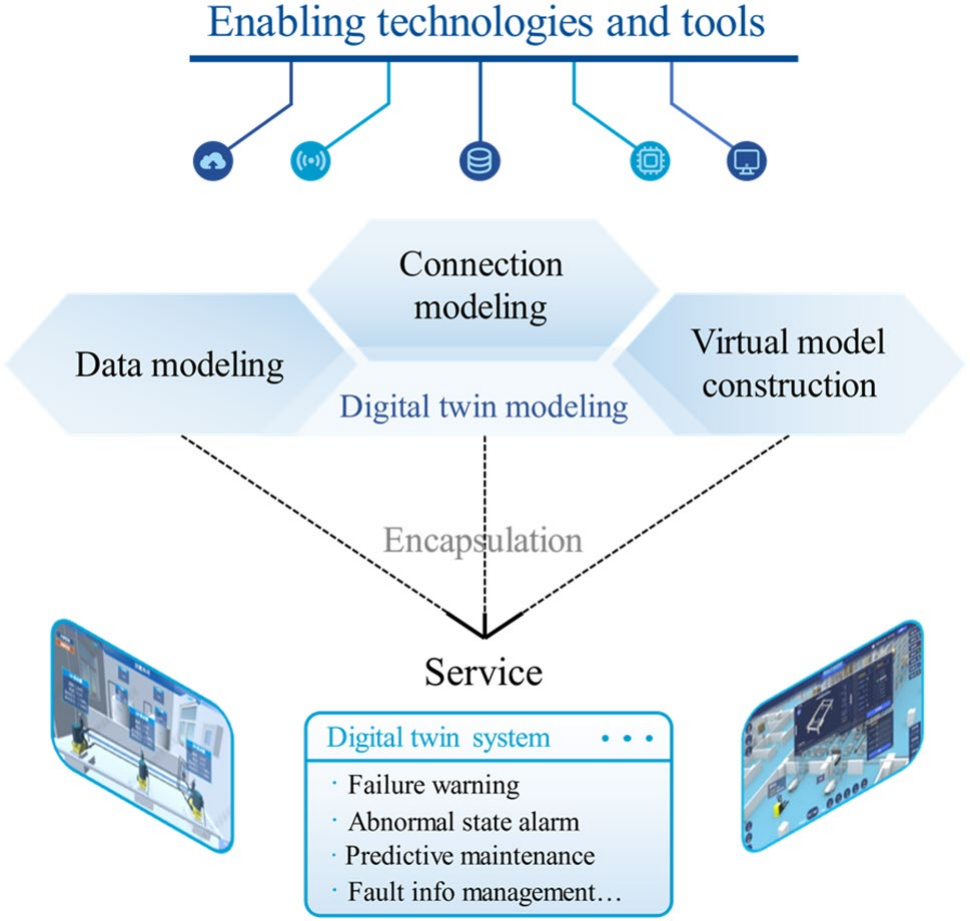
Framework of enabling technologies and tools for DT modeling, and DT systems for PHM.

After surveying status quo at application layer for RQ1 and analyzing the DT model at theory layer for RQ2, this section advances to implementation layer to discuss RQ3 “How implements DT in PHM?”. To fulfil the advantages of DT theory into practical benefits, it is necessary to explore enabling technologies and tools to parameterize DT model based on its mechanism and actual application status. Meanwhile, the DT system is imperative in DT-PHM implementation. Therefore, we first establish framework of enabling technologies and tools for DT modeling, and DT systems for PHM as presented in Fig. 11. As the elaboration for the framework, later subsections detail enabling technologies and tools used in DT-PHM from DT data modeling, connection modeling, and virtual model construction, as well as DT system offering PHM services for practical needs.

### Enabling technologies and tools of DT data modeling

**Figure 12:**
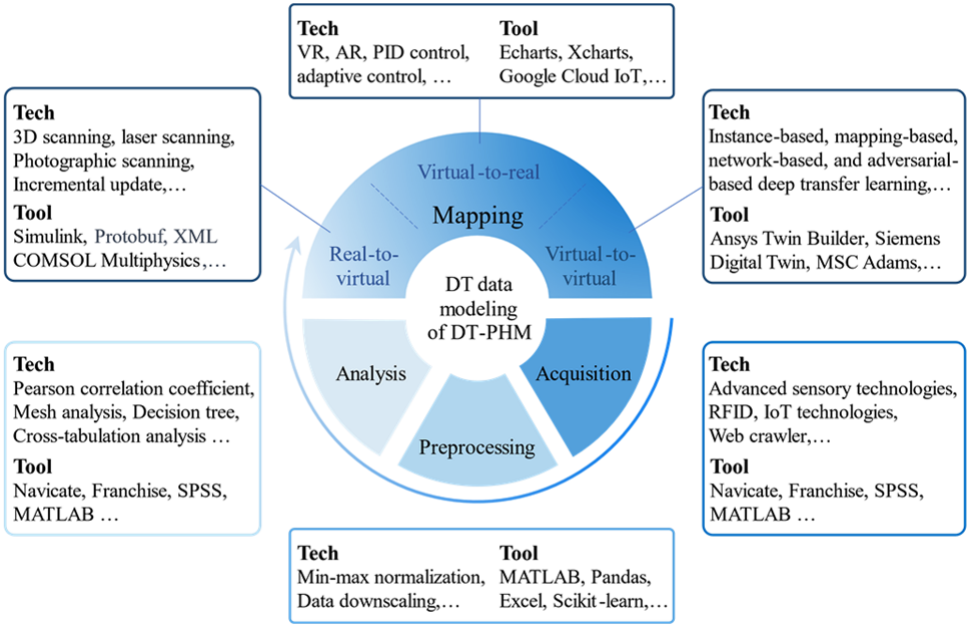
Enabling technologies and tools of DT data modeling for PHM.

DT data modeling exploits and extends physical entity data to reveal relationships between operational states and abnormal factors. For example, the data modeling linked machine state quantities to its failure modes, facilitating abnormal state assessment and abnormality localization^54^. Data acquisition, preprocessing, analysis, and mapping comprise DT data modeling^55^. Except data mapping, data acquisition^56^, preprocessing^57^, and analysis^55^ were researched and relevant enabling technologies and tools used in DT-PHM studies are summarized in Fig. 12. Data mapping establishes multiple data channels and feedback loops between virtual and real world, providing DT-driven approaches advantages over data-driven and model-driven ones. Therefore, based on theoretical analysis in subsection "DT data to PHM", this subsection elaborates on enabling technologies and tools of data mapping in DT data modeling, addressing RQ3 from the data perspective.

#### Enabling technologies and tools of data mapping from real to virtual

Real to virtual data mapping abstracts and reorganizes the physical system data into coherent and logical representation in virtual space. It includes static and dynamic mapping.

1. Static mapping from real to virtual.
  The static mapping relates the correlation between physical entity and its models in terms of static or inherent parameters which are not influnced by outside. Point cloud technology^58^ digitalizes the surface shape and features of physical entities in three-dimensional space, realizing the mapping of geometric data from real to virtual. In^59^, multispectral point cloud data on device surface was extracted and classified by machine learning methods to realize the suface defect detection. 3D scanning technology is a approach to generate point cloud data. 3D scanning was used to integrate geometrical data of appearance and structure into DT models of the lathe without disassembly, promoting fault visualization^60^. Figure 12 presents other technologies while inaccessible for mapping physical static data like material and quality, which remain constant under normal conditions but impact the depth of fault analysis. For data mapping tools, Simulink builds mathematical models of physical systems that incorporates static physical features or attribute parameters as inputs. Accordingly, parameters like resistance, capacitance, and inductance were integrated into DT model by Simulink for fault diagnosis and prediction of the power system^61^.
2. Dynamic mapping from real to virtual.
  Driven by real-time physical data, dynamic mapping updates the operational state and action, events as well as environment virtual models. Failures often coincide with abnormal changes of state and action, which manifest as deviations in physical parameters. In virtual space, the transmitted physical data is parsed and mapped to the corresponding parameters of the model. Protobuf (Protocol Buffers)^62^ serializes the real-time collected physical data into binary format for high-performance computing scenarios. In industrial automation, the sensor data of temperature, pressure, and vibration is serialized in real-time as Protobuf messages and parsed by analytical model for abnormal state analysis^63^. Real-time collected physical data can be tagged by XML (Extensible Markup Language). Jiang et al.^64^ used XML to define the data structure of various performance metrics in the engine model, thus enabling the annotation of anomalous actions reflected as discrete points in model simulation. Additionally, appropriate data mapping techniques are required to ensure model reliability due to changes in production events and external environments. Incremental update technology captured new machining tasks and variations in working conditions of the lathe to progressively improve model’s reliability on diagnosis^60^. Other technologies and tools available are presented in Fig. 12.

#### Enabling technologies and tools of data mapping from virtual to real

Data mapping from virtual to real enables the control or optimization of physical entities according to schemes or commands generated by DT models in virtual space. It includes static and dynamic mapping as well.

Static mapping from virtual to real.

Static data mapping visualizes the analysis and reasoning of DT models, by which troubleshooting reports, operation optimization solutions, maintenance strategies, etc., can be derived for physical entities. Figure 12 presents related tools that can be used on different platforms. Echarts is a JavaScript-based open-source data visualization chart library. By visualizing operation status and performance trends from DT model prediction, Echarts helps find failures promptly on the web side. On the client side, Huang et al.^65^ utilized Xcharts of Unity3D to color abnormal area on the geometric model and label it with data model-based calculations. Based on analytical results of DT models, virtual reality (VR) and augmented reality (AR) technologies can simulate potential failure scenarios to optimize O&M strategies, preventing immeasurable damage in real-life situations.

(2) Dynamic mapping from virtual to real.

Dynamic data mapping corrects physical entity's abnormal states and actions by data commands or decisions from DT models, bringing the evolutionary process between the states and actions back on track^54^. Control theory addresses how input affects output to achieve stable, fast, and accurate operation of a physical system^66^. Figure 12 illustrates corresponding technologies like PID control^67^, adaptive control^68^, and model predictive control^69^, which design appropriate controllers and algorithms by the features and goals of physical entities. Thus, data information from DT models can be transformed into effective regulations for abnormal situation. These regulations can also be compiled into executable files and deployed on cloud by platforms like Google Cloud IoT, AWS IoT^70^, and Microsoft Azure IoT^71^. However, current technologies and tools for virtual-to-real data mapping are limited to static presentations of fault detection, diagnosis, and prediction outcomes.

#### Enabling technologies and tools of data mapping from virtual to virtual

Virtual-to-virtual data mapping refines DT models by their interactions and feedback. Transfer learning is a recommended technology to achieve virtual-to-virtual data mapping. Transfer learning technologies can be categorized into four groups: instance-based, mapping-based, network-based, and adversarial-based^72^. These technologies use knowledge from one domain to improve learning efficiency and performance in another domain. For example, physical models simulate the physical characteristics, while rule models describe fault rules and logic. Using simulation data from physical models as the source domain and rule models as the target domain, network-based deep transfer learning can enhance fault analysis capability of DT model. In practice, the four groups of transfer learning can be combined to achieve better results. Meanwhile, Fig. 12 shows tools like Ansys Twin Builder^73^ and Siemens Digital Twin^74^ that support virtual-to-virtual data mapping by user-friendly interfaces to establish inter-model data connections.

### Enabling technologies and tools of DT connection modeling

**Figure 13:**
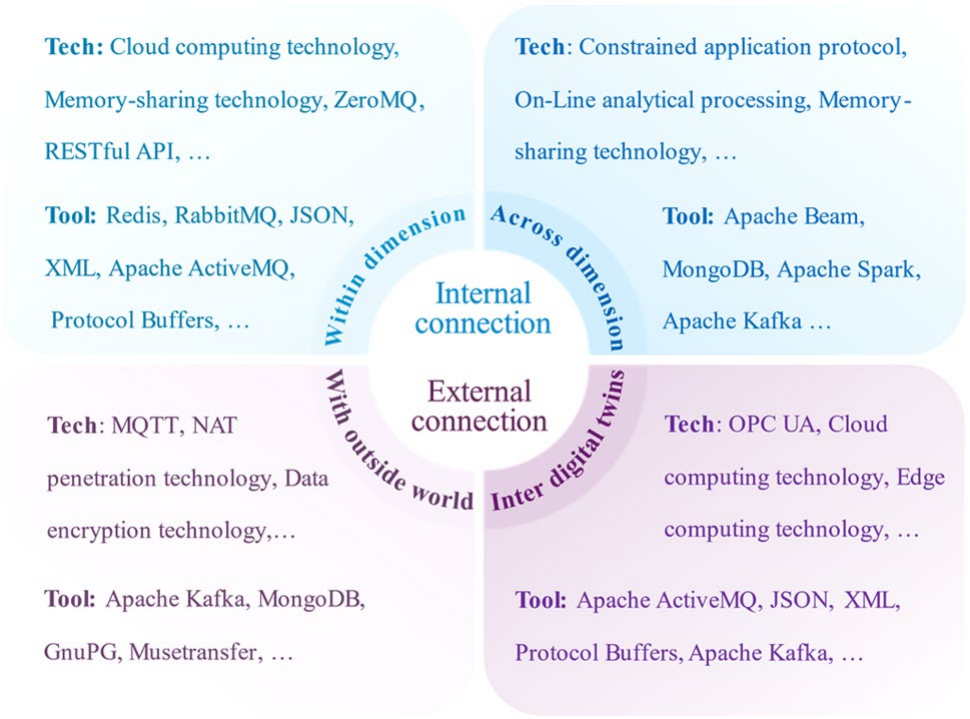
Enabling technologies and tools of DT connection modeling for PHM.

Internal connections integrate sub-models and data modules for improved simulation of physical operations. External connections facilitate comprehensive fault evaluation and reasoning by considering outside factors. Based on DT connection theories in subsection "DT connection to PHM", this subsection investigates the enabling technologies and tools of DT connection modeling as shown in Fig. 13, resolving RQ3 from internal and external connection perspectives.

1. Internal connection modeling within dimension
  Mechatronic-hydraulic fusion consolidates information that was scattered across disciplines, enabling a holistic view of the equipment. Article^48^ used memory-sharing technology for connection modeling and integrated data from disparate dimensional models, such as mechanical motion data, electrical measurement data, etc. The integrated data were analyzed by cloud computing technology for fault prediction. When sub-models access the integrated data simultaneously, distributed locks of Redis^75^ solve concurrent access issues. To simplify connection complexity and enhance data delivery between sub-models of different disciplines, dimensions, and functions, ZeroMQ^76^ and RabbitMQ^77^ are recommended.
2. Internal connection modeling across dimension
  Mechanical and electrical models gather vibration, power, and electrical signal data for fault analysis. These analyses inform the control model and enable fault correction. The connection between these functional models and data in a DT model can be established by Apache Beam based on constrained application protocol (CoAP). Apache Beam^78^ handles stream and batch data efficiently. CoAP^79^ supports fast response and real-time communication.
3. External connection modeling between DTs
  In clustered manufacturing, the DTs of multiple production units share data by connections to optimize resource utilization and reduce fault analysis costs during operation and maintenance. Some beneficial technologies and tools for DT-clustered manufacturing are presented in Fig. 13. As an open communication protocol, OPC unified architecture (OPC UA)^65^ secures the interconnection between DTs and streamlines the communication architecture to lower the data integration cost. Apache ActiveMQ^80^ supports clustered deployments, improving the communication fault tolerance of DTs. It also provides monitoring and management capabilities, enabling real-time fault analysis.
4. External connection modeling to outside world
  DTs can intelligently perceive and respond to external fault-related changes by connecting with the outside world. Message queuing telemetry transport (MQTT)^81^ enables continuous long-term connectivity, ensuring an active communication channel for the DT with the external environment, and facilitating real-time monitoring and response to external events. The data transmission also requires security. Network address translation (NAT) penetration technology^82^ encrypts and authenticates data packets to improve data transmission security. In Fig. 13, tools like Apache Kafka^83^ guarantee real-time, reliable, and secure data transmission with the outside world, making it a recommended option for DT connection modeling.

### Enabling technologies and tools of DT virtual model construction

**Figure 14:**
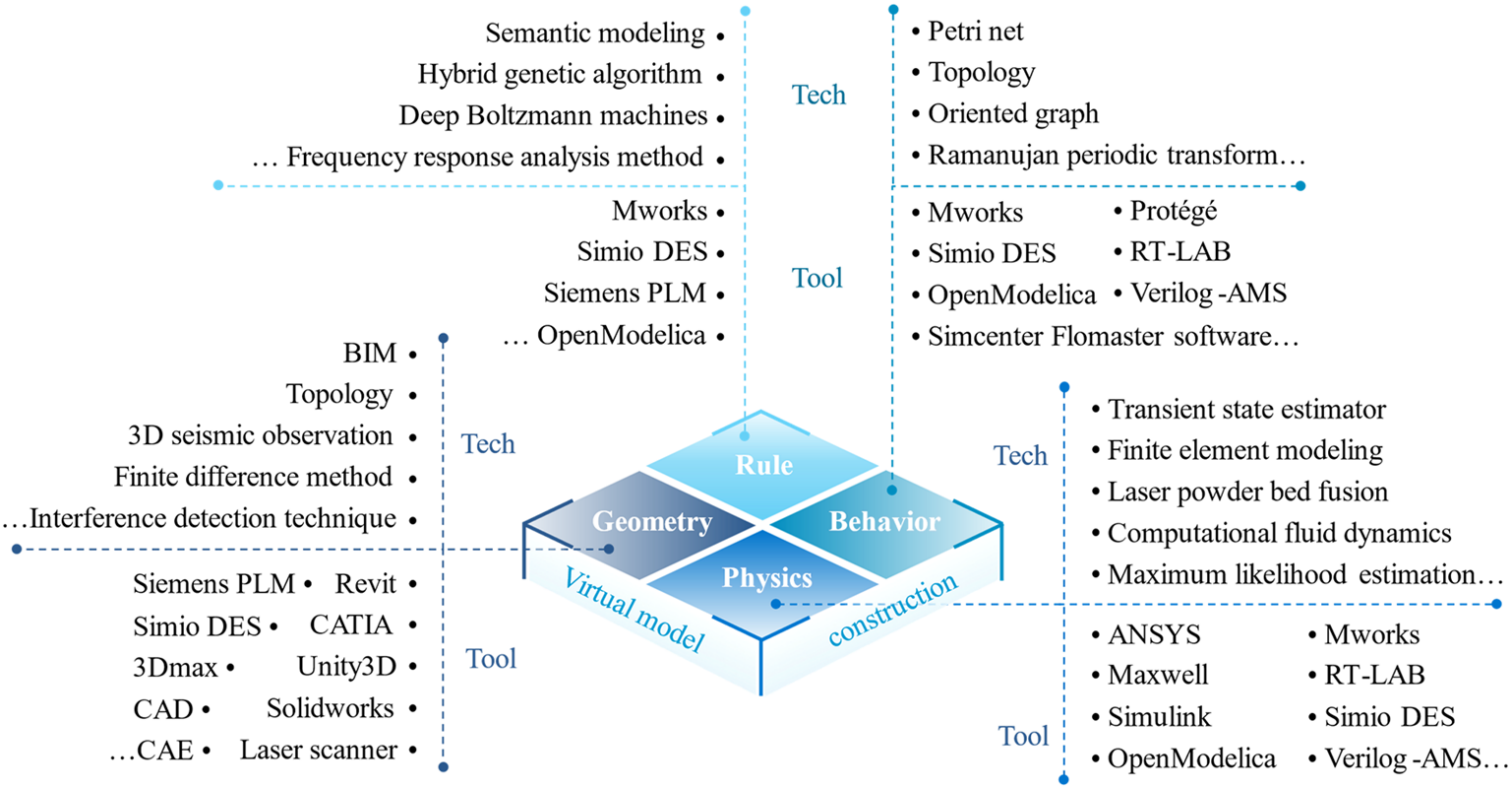
Enabling technologies and tools of DT virtual model construction for PHM.

A virtual model with multiple dimensions provides comprehensive information and optimizes DT’s capabilities to faults through the interaction among sub-models. Adequate technologies and tools for virtual model construction are essential to meet specific needs and scenarios. After discussing DT virtual model at theory layer in subsection "DT virtual model to PHM", this subsection proceeds with the enabling technologies and tools for constructing virtual models (as depicted in Fig. 14), explaining RQ3 from the perspectives of four modeling dimensions: geometry, physics, behavior, and rule.

1. Geometric modeling
  Accurate geometric modeling enhances troubleshooting efficiency by precisely locating faults on the model. Finite difference method^84^ divides the object into cubic units and calculates geometric properties within them, describing the holistic geometric characteristics precisely. Using the finite difference method, we can accurately simulate the physical properties embedded in geometric cubic units, compare them with actual observation data, detect abnormal states of devices, and predict potential failures. However, over-complex geometric modeling degrades modeling efficiency. Topology^85^ enables objects with sophisticated shapes and structures to be discretized into simple elements, facilitating the modeling process while yielding complete structural information. Topology-based techniques can adjust the modeling structure according to the needs of fault diagnosis, making it easier to detect and analyze faults. For example, optimizing the model's topology can make fault features more prominent, thereby improving the accuracy of fault diagnosis. Topological optimization can also help extract important features of objects, further assisting in data analysis and decision-making during the fault diagnosis process. Meanwhile, complexity-induced costs limit application scope and feasibility. In the building industry, Revit with building information modeling (BIM) technology minimized design costs while optimized building fault assessment^86^. Interference detection technique can be used to simplify steps for detecting abnormal events during the assembly process of complex instances in manufacturing industry.
  In Fig. 14, professional 3D modeling tools like Solidworks produce highly accurate 3D models, making DT model realistic and credible in engineering practice. Siemens PLM^87^ presents rich geometric modeling functions to create complex mechanical components, which were used in the manufacturing process simulation to predict possible faulty points.
2. Physical modeling
  Physical modeling digitally represents the physical properties of real systems in various disciplines, allowing DT models to characterize faults closer to the real situation. The modeling requires the appropriate technology based on the physical characteristics and application requirements of the actual system. For fluid dynamics systems, computational fluid dynamics (CFD) modeling^88^ precisely depicts fluid movement and heat transfer processes to optimize performance against exceptions and monitor operational faults. Finite element modeling (FEM) is for structural analysis by dividing structures into manageable cells to approximate mechanical properties, enabling numerical failure simulation of complex systems. Accurate FEM-based simulation of physical structure’s mechanical behaviors under various conditions yields precise benchmark data for comparing with real-time monitoring, aiding in identifying anomalies or faults. Additionally, four-order Runge–Kutta numerical integration method^89^ can integrate diverse differential equations, supporting the modeling of different abnormal scenarios involving various disciplines like mechanics, electronics, and thermodynamics. By applying this method iteratively at each time step, we can solve multiple differential equations simultaneously, enabling a more comprehensive consideration of various system factors and a more accurate capture of anomaly occurrence and evolution.
  Figure 14 presents that software such as RT-LAB, Maxwell, and ANSYS deliver accurate physical characterization while enable rapid modeling. Gas pressure regulator was modeled by ANSYS conveniently employing CFD and FEM to simulate its operation in a high temperature environment. This allowed real-time monitoring of properties like stress, deformation, and temperature for DT model to detect faults and perform predictive maintenance^90^.
3. Behavioral modeling
  Behavioral modeling depicts how a physical entity operates and interacts with the external environment. In^91^, oriented graph technology constructed the topology of machine production behaviors such as start, run, and stop to diagnose the abnormal behavioral states. It can be extended to represent the control and regulation process of the production line with a transfer probability matrix of behavioral states between machines. By monitoring the behavioral state transfer, production faults can be detected in time. Meanwhile, oriented graph technology provides a clear representation of the relationships and interactions among components in the equipment, facilitating a better understanding of the system's behavioral characteristics. This helps identify abnormal behavior paths of components in the equipment, thereby optimizing fault diagnosis strategies.
  For modeling of interaction behavior, CPN tools can construct Petri nets to represent concurrent and synchronized interaction behaviors between multiple machines on a production line and detects abnormal states during the interaction^92^. Mworks^93^ can develop steady-state, dynamic, and faulty behavior models during O&M, and integrate them into a reliable DT model. Physical models, such as hydrodynamic and heat transfer models, can also be incorporated by Simcenter Flomaster^94^ during behavior modeling to capture physical factors causing abnormal behaviors.
4. Rule modeling
  Rule modeling realizes fault analysis and decision-making during model evolution driven by mining rules and empirical knowledge. Automated modeling efficiently addresses tedious tasks and perceives variations of complex systems by algorithms and tools. Machine learning algorithms automatically uncover patterns and regularities hidden in the data and improve accuracy without manual intervention. Paper^95^ integrated self-encoder, convolutional neural network, and gated recurrent unit into a rule model with enhanced feature extraction and sequence modeling. This effectively reflected spatiotemporal rules and optimized fault prediction accuracy. Furthermore, by integrating neural network modules based on the structures and operational characteristics of physical objects, the rule model can be further optimized, effectively predicting behavioral patterns that deviate from normal operating modes in both time and space. As a multi-domain modeling tool, Simio DES^96^ automates rule modeling by input and dynamically updates model parameters based on fault feedback. Other usable technologies and tools are shown in Fig. 14.
  Current machine learning-based rule modeling technologies excel at processing large-scale and high-dimensional data, but lack transparency in their decision-making process. To enhance interpretability, physical attributes and behavioral features should be incorporated during model training and visualized on geometric models. Unfortunately, the seamless integration of multidimensional models is currently hindered by the lack of available technologies and software tools.

### DT system for PHM

**Figure 15:**
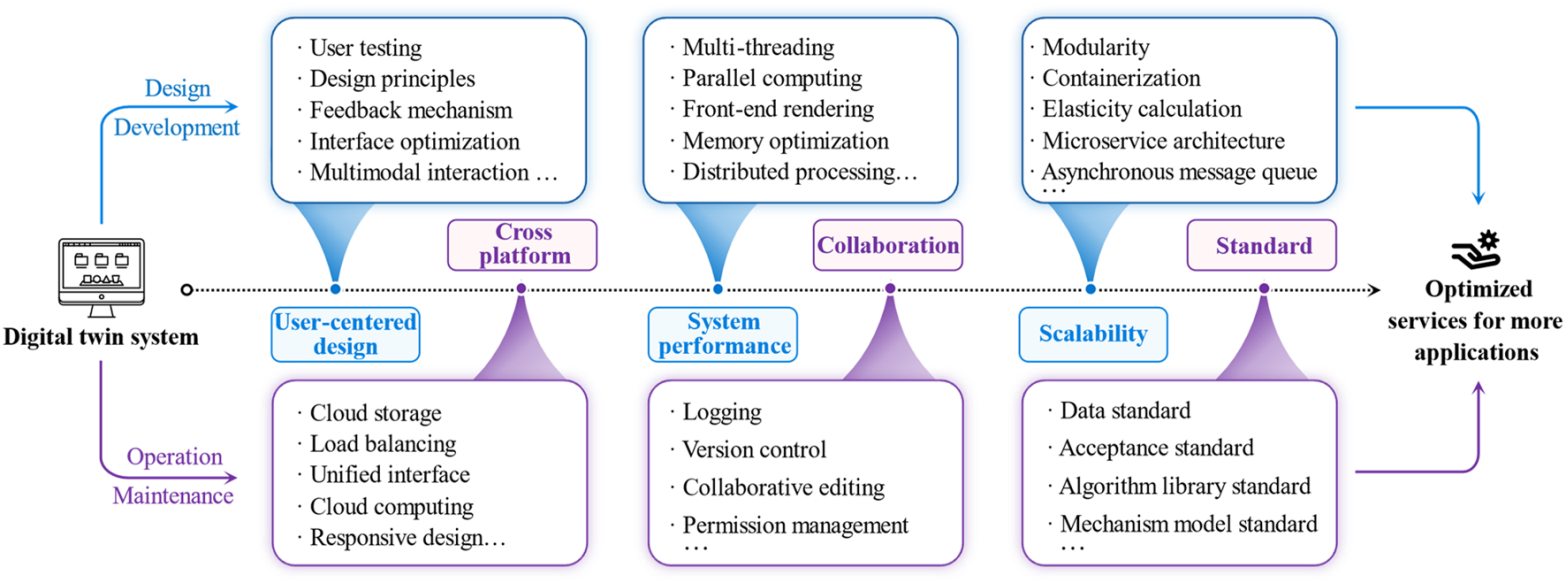
Perspectives for DT system to provide optimized service.

Based on the previous discussion of this paper, we can see that current DT-PHM studies have achieved a wide range of research outcomes from application, theory, to implementation. Remarkably, it is the DT system, which not only integrates technologies and tools, but also encapsulates DT models and their modeling into services, directly benefiting users and driving intelligent upgrades in practical applications. Out of 167 reviewed papers, 13 developed DT systems, which provided services primarily for machines and its modules. These services restricted to production status monitoring in manufacturing, anomaly alerts in the energy sector, satellite abnormity management, and building equipment overheating alarms. The complexity of the entire manufacturing system challenges the system development. Just two articles developed DT systems for troubleshooting^24^ and abnormality handling^54^ of shop floors. Meanwhile, current research lacks summary and analysis of DT systems for PHM, which hinders its deployment and optimized services for more industrial objects in various fields. Therefore, as shown in Fig. 15, this subsection studies the design, development, operation, and maintenance of the DT system for PHM from various perspectives to answer RQ3.

1. Design
  Designing a DT system requires considering user needs and experience while ensuring room for adjustment and expansion based on feedback. As shown in Fig. 15, user-centered design (UCD) provides multiple views in design to prioritize users and enhance usability. For instance, monitoring information visualization and failure analysis toolkit can be pre-customized and configured through user testing. Based on the design principles of accessibility, the optimized system interface can present simulated failure scenarios and responses through multimodal interactions like voice, gesture, and touch. Effective user feedback mechanisms empower users to refine troubleshooting methods and strategies based on system insights. Therefore, UCD underpins an efficient, reliable, maintainable, and scalable system that meets user needs in monitoring, troubleshooting, or maintenance. Only one reviewed paper^97^ provided a design template of a machine troubleshooting system, but customizability and feedback were not considered.
2. Development
  Design guides development while development realizes design. By providing convenient development environment and toolkits, Unity3D, Siemens PLM, and Mworks were used in development. Besides mature game engines and simulation software, DT system’s performance in addressing faults can be improved from the following points, as depicted in Fig. 15. Multi-threading enables simultaneous handling of multiple fault analysis tasks, processed and computed in a distributed and parallel manner. Memory optimization frees up diagnosis and prediction computing resources on the system back-end, while front-end rendering relieves the server burden for real-time monitoring information visualization.
  Scalability requires attention in development as well. Based on microservice architecture, the system can be deconstructed into independent and reusable modules, such as data acquisition and processing, status monitoring, fault detection and warning. Modules are containerized for easy deployment and management. Asynchronous message queue achieves module decoupling by delivering tasks asynchronously, while elastic computing automatically adjusts computing resources based on system load changes. These enhance system scalability in development, ensuring adaptability and feasibility to changing failure scenarios.
3. Operation
  DT systems are no longer restricted to PC-based clients, but also operating on webs^54^, or VR/AR devices^98,99^. Cross-platform compatibility enables DTs to run on multiple operating systems and hardware. Figure 15 presents that cloud computing and storage with load balancing and unified interfaces support the system's resource requirements across different platforms. Collaboration across platforms enhances fault analysis efficiency and accuracy by seamlessly integrating modules and services. Permission management and copyright control assure system security and consistency, by which co-editing and logging can facilitate defective improvement and troubleshooting in system operation.
4. Maintenance
  Maintenance of DT systems can be regulated by establishing standards as shown in Fig. 15, which improves system quality and maintainability. Standards of data, mechanism models, algorithm libraries, and acceptance need considerations in DT systems. Data standards standardize the process of DT data modeling, reducing data management costs. Mechanism model standards specify the expression, encapsulation, and interface of the mechanism model to confirm the DT model's correctness and consistency during system maintenance. Algorithm library standards reduce algorithm duplication and improve reuse of fault analysis algorithms. Acceptance standards regulate system quality, which guides system maintenance. However, the above standards are not currently implemented in DT systems.

## Challenges and opportunities

This paper presents a thorough review of DT in PHM from the insights on the faults of industrial assets. Data statistics and analytics are provided to address three RQs (Where, What, and How) from DT application, theory, to implementation for PHM. Accordingly, some challenges and opportunities are expounded as follows:

### DT application in DT-PHM

Current DT-PHM studies emphasizes fault detection of equipment and its components in the manufacturing and energy sectors. Other areas such as information, communication, and transportation are rarely addressed, while emerging areas of disciplinary convergence such as biomedical engineering have no corresponding research. Studies in manufacturing and energy sectors only analyze component faults to infer overall equipment health. However, a comprehensive evaluation requires consideration of internal and external factors based on structure and component relationships of physical entity. Furthermore, current research lacks fault analysis, handling, and faut information management after the fault is found. A full lifecycle information management system can provide a data and knowledge base for post-failure measures by comprehensively managing status and performance indicators of physical entities.

### DT model of DT-PHM

Present DT models fail to incorporate multi-source heterogeneous data and provide multiple dimensions of observation indicators for the analysis of complex fault scenarios. Image and video data can construct geometric models for deformation and damage analysis. Sensor data and logs can describe physical characteristics and behavior to simulate motions and predict performance and lifetime. Empirical knowledge data can be integrated to rule models to evaluate fault impact. By integrating sub-models and data together, the DT model underpins an intelligent monitoring system that diagnose faults and perform predictive maintenance in a timely manner.

### DT modeling of DT-PHM

In practical DT data modeling, integrating heterogeneous data from different sources challenges processing and analysis due to different time stamps, sampling rates, and data structures. Data alignment and matching techniques are needed in data processing and analysis of DT-PHM research, which enables accurate comparisons and analyses on the appropriate temporal and spatial scales.

In large-scale systems, faults often involve the interconnection of multiple components or subsystems. Therefore, multi-dimensional model construction requires knowledge from multiple subject areas, such as mechanical engineering, electrical engineering, and computer science. There is a lack of software tools for formally representing multidisciplinary knowledge to construct multidimensional models. Meanwhile, integrating knowledge and heterogeneous data to accurately characterize faults remains a technological challenge today.

Existing connection modeling technologies rely mainly on data transmission and communication, but appropriate software platforms are inaccessible for rapidly establishing connection networks within and between DTs. Failures may occur simultaneously and cross-influence. Hence, the construction of a connected network of DTs through drag-and-drop topology visualization is a promising direction for investigating the root cause and impact of faults.

### DT system of DT-PHM

Like other DT research fields, DT systems in DT-PHM studies are mainly developed by 3D game engines and simulation software. They serve primarily for entertainment and engineering simulation in specific areas but have limitations in some industrial cases. For instance, an industrial machine's failure can result from factors such as component wear, improper lubrication, unstable power supply, and operator misuse, which are beyond the scope and capability of these software. In addition, issues such as licensing, cross-platform versatility, and version compatibility also pose challenges.

## Conclusions and future work

Since its introduction in aerospace and subsequent development, the concept and application of DT have rapidly advanced in multiple fields. Along with this, DT-PHM research is in high gear as a new direction for the expansion of DT. To fill the research gaps in the existing DT-PHM reviews, this paper is problem-oriented with three RQs in DT-PHM research from insights on the faults of industrial assets and aims to address (1) “Where applies DT in PHM?” at application layer, (2) “What underpins DT in PHM?” at theory layer, (3) “How implements DT in PHM?” at implementation layer. Therefore, we conducted a systematic review of 167 publications related. The main contributions of this paper are summarized as follows:

- Provide a comprehensive analysis of DT application in PHM from the application field, aspect, and hierarchy.
- Explore data, connections, and virtual models of the DT model to unveil DT’s core and mechanisms in PHM.
- The systematic framework of the enabling technologies and tools for DT modeling, as well as DT systems for PHM is established.

Although DT- PHM research is rising, present studies in this area are still in the early stages of rapid development. Challenges remain to be addressed to enhance the practical capabilities of DT in PHM. For example, a universal software platform is demanded for full process fault analysis from detection to handling for industrial assets. Accordingly, this paper provides a reference for researchers to explore DT-PHM in future studies.

## Data Availability

The datasets used and/or analysed during the current study available from the corresponding author on reasonable request.
